# The role of ferroptosis in intervertebral disc degeneration

**DOI:** 10.3389/fcell.2023.1219840

**Published:** 2023-07-27

**Authors:** Chunyang Fan, Genglei Chu, Zilin Yu, Zhongwei Ji, Fanchen Kong, Lingye Yao, Jiale Wang, Dechun Geng, Xiexing Wu, Haiqing Mao

**Affiliations:** ^1^ Department of Orthopaedic Surgery, Orthopaedic Institute, The First Affiliated Hospital, Suzhou Medical College, Soochow University, Suzhou, Jiangsu, China; ^2^ Department of Pain Management, Zhejiang Provincial People’s Hospital, People’s Hospital of Hangzhou Medical College, Hangzhou, Zhejiang, China

**Keywords:** intervertebral disc degeneration, ferroptosis, lipid peroxidation, antioxidant system, reactive oxygen species, epigenetics

## Abstract

Nucleus pulposus, annulus fibrosus, and cartilage endplate constitute an avascular intervertebral disc (IVD), which is crucial for spinal and intervertebral joint mobility. As one of the most widespread health issues worldwide, intervertebral disc degeneration (IVDD) is recognized as a key contributor to back and neck discomfort. A number of degenerative disorders have a strong correlation with ferroptosis, a recently identified novel regulated cell death (RCD) characterized by an iron-dependent mechanism and a buildup of lipid reactive oxygen species (ROS). There is growing interest in the part ferroptosis plays in IVDD pathophysiology. Inhibiting ferroptosis has been shown to control IVDD development. Several studies have demonstrated that in TBHP-induced oxidative stress models, changes in ferroptosis marker protein levels and increased lipid peroxidation lead to the degeneration of intervertebral disc cells, which subsequently aggravates IVDD. Similarly, IVDD is significantly relieved with the use of ferroptosis inhibitors. The purpose of this review was threefold: 1) to discuss the occurrence of ferroptosis in IVDD; 2) to understand the mechanism of ferroptosis and its role in IVDD pathophysiology; and 3) to investigate the feasibility and prospect of ferroptosis in IVDD treatment.

## 1 Introduction

Low back pain (LBP) is the primary global cause of absenteeism from work and disability affecting a total of 637 million people (Vos et al., 2017; GBD 2016 Disease and Injury Incidence and Prevalence Collaborators, 2017; James et al., 2018; GBD 2017 Disease and Injury Incidence and Prevalence Collaborators, 2018) In the United States, annual spending on managing patients with LBP is estimated to exceed $100 billion (Knezevic et al., 2021). Research has shown that intervertebral disc degeneration (IVDD) is a key factor leading to LBP (Francisco et al., 2022). However, current IVDD treatment can only relieve patient’s symptoms through drugs or surgery. Conservative treatment may result in the patient’s dependence on drugs while the pain relief is insufficient. Similarly, surgical treatment is highly invasive and usually requires a long postoperative recovery along with a high recurrence rate (Andersson, 1999). Therefore, it is necessary to find new guidance to promote IVD regeneration.

**FIGURE 1:**
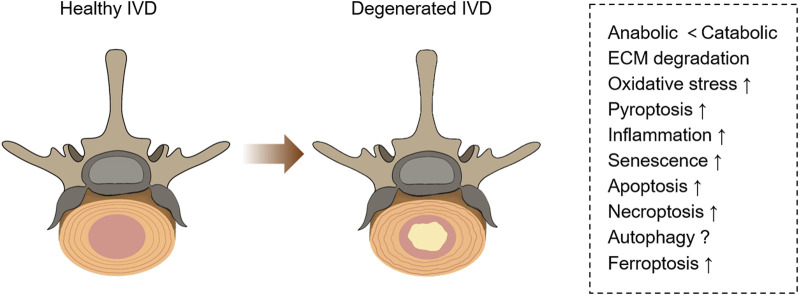
Pathological changes in healthy and degenerative IVD. Degenerative IVD will produce metabolic disorders, extracellular matrix degeneration, inflammation, and a series of programmed cell death (including necrosis, apoptosis, pyroptosis, ferroptosis, etc.).

**FIGURE 2:**
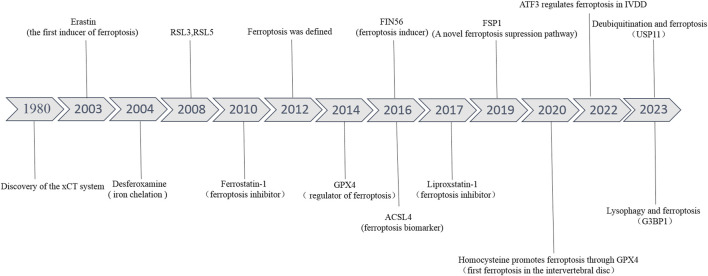
The developmental history of ferroptosis and ferroptosis in IVDD.

The related pathological changes that cause IVDD include extracellular matrix (ECM) degradation, apoptosis, senescence, and inflammation (Cazzanelli and Wuertz-Kozak, 2020) (Figure 1). During IVDD, the balance of ECM metabolism is critical for maintaining the physiological function of the intervertebral disc. Intervertebral disc cells secrete a large number of proinflammatory factors, which trigger a series of pathological reactions, leading to autophagy, senescence and apoptosis of intervertebral disc cells, resulting in decreased synthesis of aggrecan and type II collagen (Col II), and increased expression of type I collagen (Col I) and matrix metalloproteinases (MMPs). Moreover, these cytokines recruit immune cells (T cells, B cells, neutrophils, and macrophages) to further enhance the inflammatory response and accelerate disc degeneration (Phillips et al., 2015; Risbud and Shapiro, 2014). Recently, a new kind of cell death has been identified, which was named ferroptosis. Ferroptosis, described by Dixon et al., in 2012 (Figure 2), is a kind of cell death that depends on iron and reactive oxygen species (ROS) (Dixon et al., 2012; Mou et al., 2019). Lipid peroxidation, iron accumulation, ROS formation, and glutathione depletion are some of the processes involved. Ferroptosis is linked to a range of degenerative disorders, including cancer (Zhang et al., 2022), cardiovascular disease (Wu et al., 2021), orthopedic diseases (Sun K. et al., 2021), and neurological diseases (Mahoney-Sánchez et al., 2021).

In this study, we concentrated on the pathophysiology of ferroptosis and its use in IVDD in this review to offer fresh perspectives and a frame of reference for subsequent research.

## 2 Several types of regulated cell death in intervertebral disc degeneration

Cell death is a natural event that occurs in all living organisms (Green, 2019). In most previous studies, IVDD treatments were based on the prevention of cell loss due to programmed or regulated cell death (RCD). Increasing evidence shows that different forms of cell death, including apoptosis (Mirzayans and Murray, 2020), pyroptosis (Liu et al., 2021), necroptosis (Wu et al., 2020), autophagy (Galluzzi et al., 2017), ferroptosis (Chen J. et al., 2022) and senescence (Du et al., 2022), are associated with IVDD (Table 1).

**TABLE 1:** The prominent characteristics of ferroptosis, apoptosis, autophagy, necroptosis, senescence and pyroptosis in IVDD.

Cell death	Biochemical features	Morphological features	Key genes	Regulatory pathways
Apoptosis	Caspase activation; Fragmentation of oligonucleosomal DNA	Bubbling of plasma membrane; Retraction of pseudopods; Decreased cell and nuclear volume; Nuclear fragmentation; Chromatin condensation	Caspase; Bcl-2; Bax	TGF-β-mediated Smad2/3 phosphorylation signaling pathway Chen et al. (2022b); TLR2/JNK/mitochondrial-mediated signaling pathway Lin et al. (2018)
Necroptosis	The decrease in ATP; RIP1, RIP3 and MLKL activation; DAMP discharge	Plasma membrane separation; Enlargement of organelles and cytoplasm; Chromatin with a modest concentration	LEF1; RIP1, RIP3; MLKL	RIPK1/RIPK3/MLKL-mediated signaling pathway Zhang et al. (2020a)
Autophagy	Enhanced lysosomal activity; Transformation from LC3-I to LC3-II	No change in the cytoplasm; formation of double membrane autolysosomes; lack of chromatin concentration	ATG5; A TG7; Beclin 1; LC3-II; P62	AKT/mTOR-mediated signaling pathway Luo et al. (2021); PI3K/AKT/mTOR pathway Li et al. (2021b)
Pyroptosis	Release of caspase-1 and proinflammatory cytokines	Nuclear pyknosis; cell edema and membrane rupture	Caspase-1; IL-1β; IL-18	NLRP3-mediated signaling pathway Zhao et al. (2021)
Ferroptosis	GPX4 clearance; Iron accumulation; Generation of ROS and lipid peroxidation	Small mitochondria with concentrated mitochondrial membrane density; missing mitochondrial crista; ruptured mitochondrial outer membrane; the normal size of the nucleus; insufficient chromatin concentration	GPX4; SLC7A11; TFR1; NOX	xCT and Gpx4; FSP1-CoQ Zhou et al. (2022)

The most well-known RCD is apoptosis, which is often initiated by binding of the death ligand receptor, endoplasmic reticulum (ER) stress, and malfunction of the mitochondrial respiratory chain as a result of inflammation, mechanical stress, and oxidative stress (Kim and Kim, 2018). Degenerative disc expresses more Fas-L than healthy discs, indicating that degeneration may cause Fas-L-Fas binding and activate apoptosis. Pyroptosis is a form of inflammatory reaction. By recognizing specific pathogen-related molecular patterns (PRMPs) induced by exogenous pathogens and damage-related molecular patterns (DAMPs) derived from endogenous pathogens, caspase-1 is activated by a pattern recognition receptor (PRR), represented by NLRP3, which then stimulates the production of interleukin (IL)-1β, IL-18, and gasdermin-D (GSDMD), causing an extracellular IL excess and intracellular water flux leading to cell swelling and lysis (Kamali et al., 2021). ASIC1 and ASIC3 cause pyroptosis in nucleus pulposus cells (NPCs) in addition to activating the NLRP3 inflammasome by up-regulating the ROS/NF-κB signaling pathway and increasing the expression of inflammasome components (Zhao et al., 2021). Autophagy, the process by which cells self-digest and recycle damaged components, is an important mechanism for cell survival under stress conditions, especially under nutrient deprivation (Yurube et al., 2020). Autophagy is a double-edged sword for IVDD. On the one hand, autophagy can reduce the proliferation and metabolic activity of intervertebral disc cells, increase cell apoptosis and senescence, and promote IVDD. On the other hand, cartilaginous endplate stem cells can activate autophagy and inhibit IVDD by releasing exosomes to nucleus pulposus cells (Luo et al., 2021). Therefore, the role of autophagy in IVDD remains unclear. Cellular senescence is caused by DNA replication mistakes that are a byproduct of the DNA damage response. Senescent cell aggregation results in a secretory phenotype associated with aging, characterized by persistent inflammation that induces disc matrix degradation (Cherif et al., 2020). When cells lack the capability to go through apoptosis, as death ligands cannot connect to receptors, pro-survival complex I, which is composed of receptor interacting protein (RIP) 1 and others, is formed. Complex IIa or IIb is created as a result of RIP1 deubiquitination, and complex IIb formation leads to necrosis. Compression-induced NPC death in rats and herniated human NP tissues and cells have both been linked to RIP1/RIP3/MLKL-mediated necrosis in various studies.

A unique kind of cell death known as ferroptosis, which occurs in IVDD, is marked by lipid peroxidation and free iron-mediated Fenton reactions (Yang R. Z. et al., 2021). Because ferroptotic cells do not have the necessary defensive mechanisms to remove lipid peroxides, these peroxides accumulate in great numbers (Stockwell et al., 2020). Various morphological features set ferroptosis apart from other RCD processes. We have discovered that IVDD cells subjected to ferroptosis often exhibited mitochondrial shrinkage, reduced number, increased membrane density, disruption of the normal structure of the mitochondrial crest, regular nuclear size, and membrane foaming without rupture (Dixon et al., 2012; Xie et al., 2016; Yu et al., 2017; Han et al., 2020).

## 3 Mechanisms and regulation of ferroptosis

**FIGURE 3:**
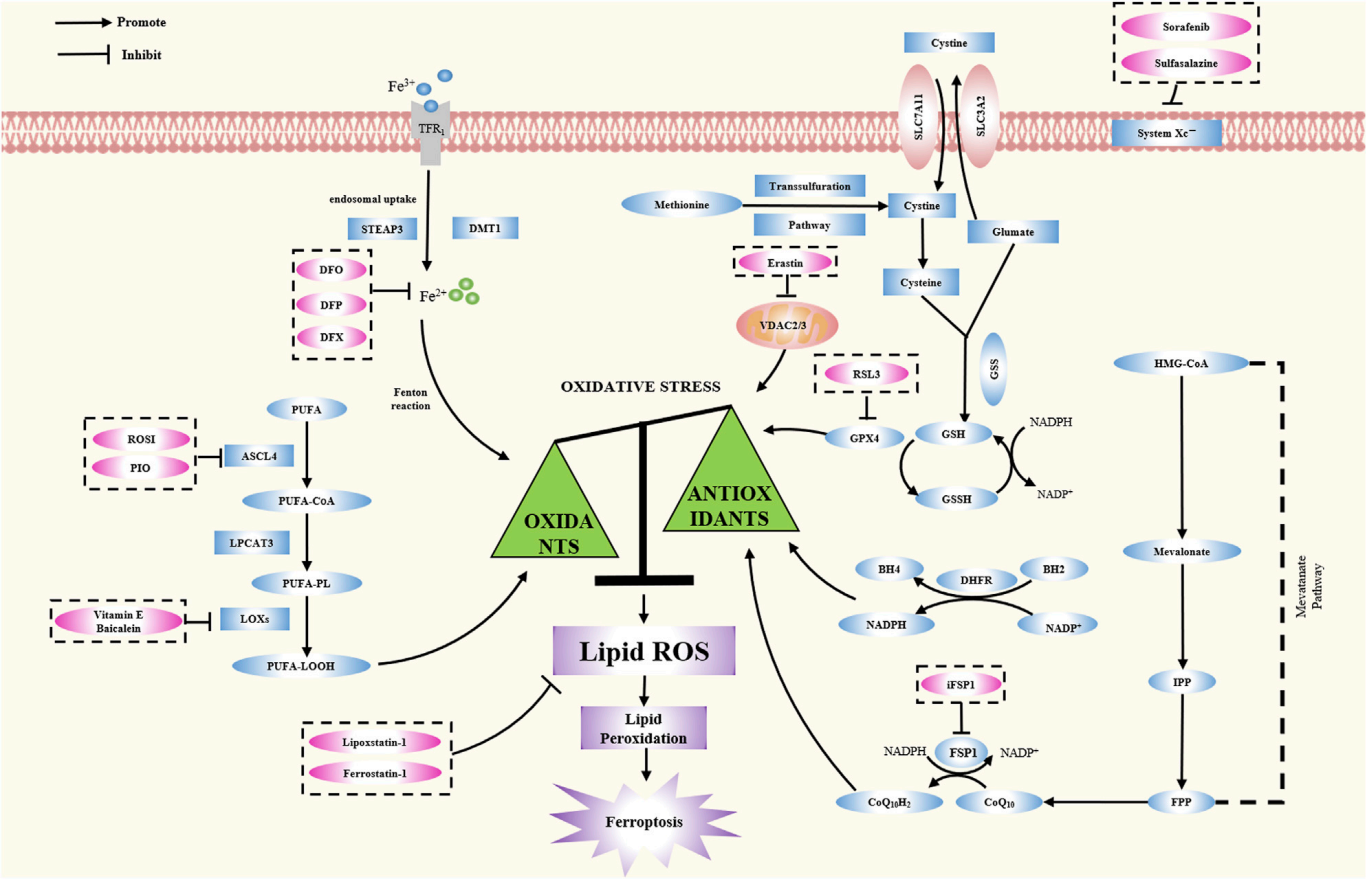
The latest reported mechanism of ferroptosis. The essential feature of ferroptosis is the imbalance between oxidative stress and anti-oxidative stress system. This article reviews the pathogenesis of ferroptosis, including its occurrence mechanism, regulation mechanism and typical inhibitors (Table 2).

Ferroptosis is driven by the imbalance between oxidation and antioxidant systems, involving ROS accumulation, lipid peroxidation, and various metabolic pathways (Figure 3). Ferroptosis is accompanied by an increase in ROS generation due to the accumulation of harmful lipid peroxides. The selective permeability of the plasma membrane is lost, and the membrane lipids become peroxidized. Studies of ferroptosis have shown various mechanisms that might initiate or influence the process. This paper reviews the important roles of iron metabolism, lipid metabolism, amino acid metabolism, and oxidative stress in ferroptosis pathogenesis.

### 3.1 Oxidation mechanisms and regulation

#### 3.1.1 The role of iron metabolism

Iron, which is involved in many critical biological activities, such as oxygen transport, DNA biosynthesis, and ATP creation, is a trace element vital to cell function (Bogdan et al., 2016). Therefore, maintaining iron homeostasis is crucial. The body obtains more than two-thirds of its iron from hemoglobin in red blood cells, with the majority of the remaining portion being stored in macrophages and liver cells (Puntarulo, 2005). Iron metabolism is divided into four components: uptake, storage, utilization, and export (Chen et al., 2021). TF/TFR binds iron very firmly and has been linked to iron intake. In most mammalian cells, iron uptake occurs through endocytosis of transferrin by specific receptors. After binding to the transferrin receptor, iron bound to transferrin in plasma can be taken up by cells via receptor-mediated endocytosis (Anderson and Frazer, 2017). The resultant complex is then transported to the endosomes, where Fe^3+^ is released in an acidic environment. Afterward, Fe^3+^ is converted to Fe^2+^ by STEAP3 (six-transmembrane epithelial antigen of prostate family member 3), which is an endosomal iron reductase (Li et al., 2020). With the help of SLC11A2/DMT1 (member 2 of solute carrier family 11), Fe^2+^ is further transported into the cytoplasm. There are three fates of iron after its intake. First, it is fed to the labile iron pool (LIP) for use (Philpott et al., 2017). Second, it can be saved in ferritin for future use. About 70%–80% of the freshly imported iron is stored in ferritin, which is the primary iron storage protein. Ferritin is divided into two subtypes, H and L, also known as FTH1 (ferritin heavy chain 1) and FTL (ferritin light chain). With its iron oxidase activity, FTH1 converts Fe^2+^ to Fe^3+^. FTL contributes to iron nucleation and mineralization (Knovich et al., 2009). Through holes in the ferritin shell, iron enters the ferritin lumen and travels to the catalytic ferroxidase core, where it is internally deposited as an iron hydride. Finally, cells can also remove iron from themselves via the output of FPN/SLC40A1. If large amounts of iron build up inside the cell, this output pathway can act as a safety valve.

Iron is absorbed primarily through the duodenum, and unabsorbed iron enters the colonic cavity, where many intestinal flora reside. Iron is essential for these bacteria, and taking iron orally can promote the growth and virulence of bacterial pathogens in the gut, leading to intestinal inflammation. In addition, gut bacteria also have the ability to transport iron, affecting iron metabolism (Seyoum et al., 2021). Iron metabolism is closely related to ferroptosis, therefore, intestinal flora affects ferroptosis. Lou et al. found that urinary phospholipid A, an anti-inflammatory metabolite of gut microbiota, attenuated ferroptosis in LPS-induced acute lung injury in mice by up-regulating the Keap1-Nrf2/HO-1 signaling pathway (Lou et al., 2023). Deng et al. found that the intestinal microbial metabolite capsiate inhibits intestinal ischi-reperfusion induced ferroptosis by activating TRPV1 (Deng et al., 2021). However, the relationship between intestinal flora and ferroptosis in IVDD has not been reported.

Atherosclerosis, hemochromatosis, anemia, and joint disease have all been linked to iron metabolism abnormalities (Dabbagh et al., 1993; Weiss and Goodnough, 2005; Vinchi et al., 2020). Low iron levels stimulate TFR1 production and suppress the expression of ferritin and FPN1, whereas high iron levels within the cells may suppress DMT1 expression in an IRE-IRP-mediated way, which is associated with ferroptosis (Rouault, 2006). Cellular susceptibility to ferroptosis is impacted by modifications in iron homeostasis regulators. It has been reported that *TF* knockout can reduce ferroptosis induced by siramesine and lapatinib in MDA-MB-231 and SKBR3 cancer cell lines (Ma et al., 2016). Loss of TFRC also prevents ferroptosis triggered by cystine deprivation or erastin, suggesting that ferroptosis is closely related to iron uptake (Yang and Stockwell, 2008; Gao et al., 2015). *FTH1-mutated* flies develop significant growth defects due to, in part, ferroptosis induction (Mumbauer et al., 2019). Knocking down *SLC40A1* enhances ferroptosis, whereas its overexpression inhibits ferroptosis (Geng et al., 2018).

Many enzymes that generate ROS need iron or iron derivatives as their active centers (e.g., lipoxygenase, cytochrome P450, and NADPH oxidase) (Doll and Conrad, 2017). Although iron has a vital function in maintaining life, iron overload is toxic, generating ROS and triggering cell death. Currently, iron chelators are the standard treatment for iron excess sickness. As classical iron chelating agents, DFO, DFP, and DFX bind to Fe^3+^ *in vivo* to form stable complexes, which can be cleared by urine or bile (Rodrigues de Morais and Gambero, 2019; Chen X. et al., 2020). Additionally, lactoferrin possesses anti-inflammatory and antioxidant properties. To some degree, human lactoferrin may function as an iron chelator by neutralizing free iron in synovial fluid (Guillén et al., 1998).

Ferroptosis may be prevented both *in vivo* and *in vitro* by using iron chelators. First discovered to counteract erastin and RSL3-induced ferroptosis, DFO is now the most extensive medication for preventing lipid peroxidation-mediated ferroptosis (Dixon et al., 2012). Deferasirox inhibits heme-induced human monocyte ferroptosis and ROS production (Imoto et al., 2018). Baicalein is a flavonoid that reverses ferroptosis in human pancreatic cancer cell lines through its free 5,6,7-hydroxyl complex with iron in a 1:1 ratio (Xie et al., 2016). Particularly, 2,2′-bipyridine isolates iron from unstable iron pools (LIPs) through its membrane permeability, chelates mitochondrial iron in mitochondria, and reduces ROS production (Chang et al., 2016).

#### 3.1.2 The role of lipid metabolism

Fatty acids (FAs) are vital building blocks of biofilm lipids and substrates for energy metabolism. FAs are primarily synthesized by acetyl-CoA carboxylase (ACAC), acetyl-CoA, malonyl-CoA, FASN (FA synthetase), ELOVL (ELOVL FA elongase), and FA desaturase. Lipid droplets, which are composed of triglycerides and cholesterol esters, store FAs that protect against palmitic acid-induced lipotoxicity by partitioning off damaged cell membranes. Oxidative pathway reactions, including FA activation, acyl-CoA transfer, and oxidation of acyl-CoA, mostly take place in mitochondria and are essential for FA use.

A major cause of ferroptosis is lipid peroxidation. Lipid hydroperoxides (LOOHs) and then reactive aldehydes, such as malondialdehyde (MDA) and 4-hydroxynonenal (4HNE), are produced during the free radical-driven lipid peroxidation, which primarily targets unsaturated FAs on the cell membrane. Lipid peroxides cause membrane disintegration by destabilizing the lipid bilayer. Toxic lipid ROS are produced when an abundance of Fe^2+^ accumulates in the cytoplasm, damaging the cells they reside in and leading to a series of morphological features of ferroptosis. As a result of the presence of highly active hydrogen atoms in methylene bridges, polyunsaturated FAs (PUFAs) are more susceptible to lipid peroxidation. The PUFAs in membrane phospholipids may combine with hydroxyl radicals to create lipid peroxides, which, in turn, promote ferroptosis (Doll et al., 2017; Yan et al., 2021). As a result of their work, ACSL4 and LPCTA3 enrich the cell membrane with sensitive PUFAs, which are necessary for the oxidation of arachidonic acid (AA) and adrenalic acid to phospholipids (PLs). Furthermore, Fe^2+^ may work with lipoxygenase (LOX) as a cofactor to accelerate the lipid peroxidation of PUFA (Yang et al., 2016). Increased LOX15 and PUFA binding in the cell membrane, caused by phosphatidylethanolamine (PE) binding protein 1 (PEBP1), is necessary for ferroptosis to occur (Wenzel et al., 2017).

LOX is a non-heme iron dioxygenase in the acyl oxidation of PUFAs to lipid hydroperoxides (Mashima and Okuyama, 2015; Shintoku et al., 2017). LOX is one of the key factors driving lipid peroxidation during ferroptosis, especially at the initial stage (Yang et al., 2016). When undergoing ferroptosis, LOX promotes ROS accumulation, lipid peroxidation, and cell death. LOX inhibitors, which thus inhibit ferroptosis (Probst et al., 2017), include selective 12/15-LOX inhibitors (Deschamps et al., 2006), pan-LOX inhibitors, selective 5-LOX inhibitors (Rossi et al., 2010), and selective 15-LOX inhibitor (Sendobry et al., 1997) (Table 2). Additionally, many LOX inhibitors can also be used as free radical catchers and terminators of lipid autoxidation free radical chain reactions to inhibit ferroptosis (Hanthorn et al., 2012; Shah et al., 2018). The absence of free radical capture in LOX inhibitors, such as ML351, may also be able to demonstrate antiferroptotic activity (Kagan et al., 2017).

**TABLE 2:** Ferroptosis inhibitors.

Name	Mechanism	References
Deferoxamine (DFO)	Bind to Fe3+	Abdul et al. (2021)
Deferasirox (DFX)	Bind to Fe3+	Jomen et al. (2022)
Deferiprone (DFP)	Bind to Fe3+	Stockwell et al. (2017)
Dexrazoxane	Regulates iron level	Fang et al. (2019)
Ciclopirox	Regulates iron level	Stockwell et al. (2017)
Baicalein	Regulates iron level	Xie et al. (2016)
2,2′-bipyridine	Regulates iron level	Dixon et al. (2012)
1,10-phenanthroline	Regulates iron level	Ye et al. (2021)
Zileuton	Inhibits 5-LOX	Yuan et al. (2016)
PD146176	Inhibits 15-LOX-1	Zilka et al. (2017)
Baicalein	Inhibits 12/15-LOX	Probst et al. (2017)
Curcumin	Inhibits 15-LOX-1	Yang et al. (2021a)
NDGA	Inhibits pan-LOX	Probst et al. (2017)
AA-861	Inhibits 5-LOX	Li et al. (2014)
CDC	Inhibits LOX	Yang et al. (2016)
H2S	Inhibits LOX	Wang et al. (2021b)
ML351	Inhibits LOX	Kagan et al. (2017)
NCTT-956	Inhibits LOX	Kagan et al. (2017)
Vitamin E	Restrains LOX PUFA oxygenation	Kagan et al. (2017)
Trolox	Restrains LOX PUFA oxygenation	Kagan et al. (2017)
D-α-Tocopherol	Restrains LOX PUFA oxygenation	Kagan et al. (2017)
tocotrienols	Restrains LOX PUFA oxygenation	Kagan et al. (2017)
NH4Cl	Inhibits lipid peroxidation	Torii et al. (2016)
Butylated hydroxytoluene (BHT)	Inhibits lipid peroxidation	Nieva-Echevarría et al. (2017)
Pepstatin methyl ester	Inhibits lipid peroxidation	Torii et al. (2016)
Fer-1	Inhibits lipid peroxidation	Dixon et al. (2012)
Lip-1	Inhibits lipid peroxidation	Friedmann Angeli et al. (2014)
Indole-3-carbinol	Inhibits lipid peroxidation	Yagoda et al. (2007)
β-carotene	Inhibits lipid peroxidation	Yagoda et al. (2007)
Butylated hydroxyanisole	Inhibits lipid peroxidation	Shimada et al. (2016)
Triacsin C	Inhibits ACSL4	Kagan et al. (2017)
Troglitazone	Inhibits ACSL4	Doll et al. (2017)
Rosiglitazone	Inhibits ACSL4	Yang et al. (2016)
MUFAs	Blocks PUFA peroxidation	Magtanong et al. (2019)
Lactate	Promotes PUFA production	Zhao et al. (2020)
Temozolomide (TMZ)	Induces system xc−expression	Sehm et al. (2016)
Ebselen	Simulates glutathione peroxidase	Matsushita et al. (2015)
β-mercaptoethanol	Reduces Cys2 to Cys (Cystine uptake)	Sha et al. (2015)
Aminooxyacetic acid	Inhibits glutaminase	Gao et al. (2015)
Compound 968	Inhibits glutaminase	Gao et al. (2015)
Allosteric GPX4 activators	Activates GPX4	Li et al. (2019a)
CDOD	Inhibits GPX4 degradation	Wu et al. (2019)
TOFA	Inhibits GPX4 degradation	Lee et al. (2020a)
N-Acetylcysteine (NAC)	Supplements GSH	Yang et al. (2014)
Selenium	Enhances the number of selenoproteins	Alim et al. (2019)
Dopamine	Increases the stability of GPX4	Wang et al. (2016)
Idebenone	Mimics CoQ10	Doll et al. (2019)
iFSP1	Inhibits the function of FSP1	Jo et al. (2022)
Diarylamine	Acts as RTA	Shah et al. (2017)
C15-THN	Acts as RTA	Conrad and Pratt (2019)
Phenothiazine	Acts as RTA	Yang et al. (2021d)
Phenoxazine	Acts as RTA	Poon et al. (2020)
tetrahydrobiopterin	Acts as RTA	Kraft et al. (2020)
Cu^II^ (atsm)	Acts as RTA	Southon et al. (2020)
Omeprazole	Acts as RTA	Mishima et al. (2020)
Promethazine	Acts as RTA	Mishima et al. (2020)
Propranolol	Acts as RTA	Mishima et al. (2020)
Rifampicin	Acts as RTA	Mishima et al. (2020)
Carvedilol	Acts as RTA	Mishima et al. (2020)
Estradiol	Acts as RTA	Mishima et al. (2020)
JP4-039	Scavenges lipid ROS	Mishima et al. (2020)
Mitoquinone	Eliminates mitochondrial ROS	Jelinek et al. (2018)
Puerarin	Inhibits ROS production and Ca^2+^ influx	Dixon et al. (2012)
KI-696	Inhibits NRF2 degradation	Dixon et al. (2012)
Alogliptin	Prevents ROS accumulation	Xie et al. (2017)
Linagliptin	Prevents ROS accumulation	Xie et al. (2017)
Vildagliptin	Prevents ROS accumulation	Alborzinia et al. (2018)
2-acetylphenothiazine	Prevents ROS accumulation	Xie et al. (2017)
DPI	Prevents ROS accumulation	Yang et al. (2020a)
GKT137831	Prevents ROS accumulation	Abou Daher et al. (2020)
GKT136901	Prevents ROS accumulation	Hwang et al. (2020)
6-aminonicotinamide	Prevents ROS accumulation	Dixon et al. (2012)
2ME	Suppresses erastin-induced ferroptosis	Dixon et al. (2012)
Cycloheximide	Affects protein synthesis	Dixon et al. (2012)
SB202190	Targets P38	Yu et al. (2015)
SP600125	Targets JNK	Zalyte and Cicenas (2022)

Fatty acyl-CoA esters are formed from free long-chain FAs by ACSL proteins, which are mostly found in the ER and mitochondrial outer membrane (OMM). Acyl-CoA synthetase long-chain family 4 (ACSL4), as a member of the ACSL family, is considered a marker of ferroptosis. AA and other lipid peroxides with PE omega-6 FAs are produced by ACSL4, which also catalyzes AA-CoA synthesis (Capelletti et al., 2020). By itself, *GPX4* knockout triggers ferroptosis; however, *GPX4* and *ACSL4* cells could develop normally, indicating that ACSL4 inhibitors suppress ferroptosis (Doll et al., 2017). ACSL4 is selectively inhibited by thiazolidinediones (TZD) (Kim et al., 2001), such as rosiglitazone (ROSI). ROSI improves intestinal barrier failure due to ischemia-reperfusion by preventing ACSL4 activation and decreasing lipid peroxidation and ferroptotic cell death (Li Y. et al., 2019). Triacsin C also has the potential to inhibit ACSL4 (Zhu et al., 2022).

Ferroptosis is triggered by monounsaturated FAs (MUFAs), as shown by Magtanong et al. (Magtanong et al., 2019), and relies on ACSL3 (acyl-CoA synthase long-chain family member 3). MUFAs do not increase GPX4 expression but prevent the accumulation of plasma membrane lipid ROS and reduce the incorporation of PUFAs into phospholipids. Lactate is a nutrient that has many regulatory effects and is associated with oxidative stress resistance and lipid biosynthesis (Chen et al., 2016). Hepatocellular carcinoma cells have a lactate-mediated ferroptosis regulation system, as discovered by Zhao et al. Via this pathway, lactic acid can enhance the anti-ferroptosis ability of HCC cells by up-regulating the production of anti-ferroptotic MUFAs (Zhao et al., 2020).

### 3.2 Antioxidant mechanisms and regulation

#### 3.2.1 Glutathione metabolism

Ferroptosis occurs when the body antioxidant and oxidation defenses cannot function properly. Lipid peroxidation, membrane damage, and ferroptosis are all results of an unbalanced environment, such as increased ROS generation or reduced antioxidant system activity (Hassannia et al., 2019). Along with the Xc^−^ system and GPX4, glutathione (GSH) serves as the major antioxidant in mammalian cells, preventing the buildup of lipid hydrogen peroxide. Solute carrier family 7 member 11 (SLC7A11) and solute carrier family 3 member 2 (SLC3A2) form the heterodimer protein complex glutamate/cystine reverse transporter system Xc^−^. While SLC7A11—a multichannel transmembrane protein—is responsible for regulating the action of the glutamate/cystine reverse transporter in the Xc^−^ system, SLC3A2 keeps SLC7A11 stable and in the right place on the membrane (Nakamura et al., 1999; Koppula et al., 2018). Besides encouraging the transmembrane interchange of external cysteine and internal glutamate, the transporter also stimulates the uptake of cysteine into the cell for its production. Glutathione consists of three amino acids: glutamate, cysteine, and glycine, which are arranged in a tripeptide structure. Cysteine is often cited as the limiting factor in glutathione formation due to its limited intracellular content. Three precursor substances (glutamate, cysteine, and glycine) form GSH by glutamate–cysteine ligase (GCL). For GPX4 to continue functioning and being expressed, GSH is required as a cofactor. GPX4 is a selenoprotein that converts active PLOOH to inactive phosphatidylcholine (PLalchol, PLOH), hence, blocking the free radical chain reaction responsible for lipid peroxidation (Hassannia et al., 2019). By employing glutathione as a substrate, GPX4 converts hazardous membrane lipid hydroperoxides into lipid alcohols, which are non-toxic. In addition to neutralizing active Fe^2+^, GPX4 may also convert H_2_O_2_ into H_2_O, preventing ferroptosis.

The destruction of the antioxidant barrier induces ferroptosis. Xc^−^ is a sodium-dependent reverse transporter that absorbs extracellular cystine in exchange for an equal amount of intracellular glutamate (Bannai, 1986; Conrad and Sato, 2012). Many cancer cells may be effectively induced to undergo ferroptosis by eliminating cystine or preventing SLC7A11 from functioning via gene ablation or pharmacological suppression. In contrast, SLC7A11 overexpression stimulates GSH production and suppresses ferroptosis in cancer cells. GSH is essential in ferroptosis because of its antioxidant properties. Many studies have demonstrated that cells are more vulnerable to oxidative stress when intracellular GSH levels are low. Evidence suggests that reduced glutathione levels promote ferroptosis (Niu et al., 2021). Inhibiting glutathione production, whether chemically or genetically, stunts tumor cell development and triggers iron-like cell death (Liu et al., 2017). In ferroptosis, GPX4 alterations have regulatory effects. In contrast to mercaptoethanol, which prevents ferroptosis by boosting GPX4 activity via increasing cystine transport into cells, heat shock protein 90 prevents ferroptosis by blocking GPX4 breakdown (Dixon et al., 2012; Wu et al., 2019).

Temozolomide (TMZ) is a lipophilic 194-Da soluble molecule and an oral imidazolotetrazine DNA-alkylating agent. TMZ is thought to be an inhibitor of ferroptosis, which acts by inducing Xc^−^ expression. In glioblastoma multiform cells induced by TMZ, Xc^−^ ubunit expression is significantly increased, GSH synthesis is increased, and ROS levels are decreased (Sehm et al., 2016). In the presence of erastin, glutamine can be broken down into glutamic acid and ammonia *in vivo* through the action of glutaminase, inducing ferroptosis. Aminooxyacetic acid, a glutaminase inhibitor, heals heart tissue injury in mice by preventing ferroptosis via blocking glutamine metabolism (Gao et al., 2015). N-Acetylcysteine (NAC) itself cannot protect GPX-deficient cells but serves as a precursor to glutathione, which then enhances the activity of the GPX4-GSH-cysteine axis and has an antiferroptotic effect (Seiler et al., 2008). Furthermore, selenium is a crucial trace element involved in mammalian life, primarily as a selenoprotein. GPX4 is an extremely significant selenoprotein. Selenium protects GPX4 from irreversible inactivation while driving GPX4 transcriptional expression, thereby inhibiting ferroptosis (Friedmann Angeli and Conrad, 2018).

#### 3.2.2 FSP1-CoQ system

Coenzyme Q (CoQ) is an endogenous isovalentyl benzoquinone molecule that transports electrons between complexes I and II and complex III in the mitochondrial electron transport chain. Eukaryotes use the mevalonate pathway to generate CoQ_10_. The mevalonate (MVA) route and its intermediate isopentenyl pyrophosphate (IPP) are crucial in cellular metabolism. Under the influence of pyrophosphate synthase (GGPS), it may create CoQ10 in addition to cholesterol, vitamin K, and heme (Holstein and Hohl, 2004). Later, CoQ10 is converted to CoQ10H2 through FSP1-catalyzed reactions. As a result of its ability to eliminate PLOOH, CoQ10H2 may halt the lipid oxidation chain reaction and prevent ferroptosis. FSP1 is a scavenger protein that resides in lipid droplets and plasma membrane, where it helps remove dangerous lipid hydroperoxides (Bersuker et al., 2018; Doll et al., 2019). According to available research, the FSP1/CoQ10/NAD(P)H route can prevent ferroptosis without involving the GSH/GPX4 pathway (Hadian, 2020).

FIN56 may function as a ferroptosis agent by activating squalene synthase (SQS) to promote the MVA pathway and, thus, cholesterol synthesis, thereby decreasing the formation of CoQ10 (Ingold et al., 2018).

In addition, idebenone, a water-soluble CoQ10 analog, mimics the antioxidant effects of CoQ10 and inhibits FIN56-induced ferroptosis without changing the level of basal lipid ROS (Shimada et al., 2016). Evidence suggests that iFSP1 prevents ferroptosis by counteracting FSP1 pro-lipid-peroxidation effects. Conversely, it may also sensitize cancer cells that were previously resistant to RSL3-induced ferroptosis by promoting ferroptosis in GPX4-KO cancer cells that overexpress FSP1 (Doll et al., 2019).

#### 3.2.3 Other antioxidant mechanisms and regulation

As a channel protein on the mitochondrial outer membrane, voltage-dependent anion channel 2/3(VDAC2/3) allows a large number of ions and metabolites to pass in and out between cytoplasm and mitochondria, which has a crucial function in preserving homeostasis maintenance *in vivo* (Wang Z. et al., 2020). Reportedly, erastin-induced ferroptosis in melanoma is suppressed by Nedd4 ubiquitination of VDAC2/3 (Yang Y. et al., 2020). The precise methods by which erastin and VDAC2/3 work to reduce NADH oxidation and induce ferroptosis need to be discovered. However, it is known that erastin does this by directly binding VDAC2/3, which, in turn, affects the permeability of the OMM (Yagoda et al., 2007).

Inhibition of ferroptosis through the guanosine triphosphate (GTP) cyclic hydrolase 1/tetrahydrobiotrexate (GCH1/BH4) pathway occurs independently of the GPX4 protein (Kraft et al., 2020; Soula et al., 2020). GCH1 is a rate-limiting enzyme for the production of BH4 from GTP (Thöny et al., 2000). BH4 is a powerful antioxidant that prevents lipid peroxidation from causing ferroptosis, but it has to be replenished by dihydrofolate reductase (DHFR). Inhibiting DHFR and GPX4 at the same time lowers BH4 synthesis and increases ferroptosis sensitivity. By encouraging the conversion of phenylalanine to tyrosine, BH4 plays a role in the production of coenzyme Q10, which, in turn, regulates ferroptosis.

Transfer of electrons by NADPH oxidases (NOX) in biofilms leads to the production of superoxides. NADPH is required for ferroptosis because it serves as a source of ROS and is used to produce DPP4-NOX compounds (Kang et al., 2019). After knocking down *NOX4*, there is a dramatic reversal of ventricular remodeling due to an improvement in iron overload (Chen et al., 2019). *P53* deletion hinders the nuclear accumulation of dipeptidyl-peptidase 4 (DPP4), which consequently promotes membrane-associated DPP4-mediated lipid peroxidation by interacting with NOX1, resulting in ferroptosis in colorectal cancer. Through the reduction in ROS production by preventing DPP4 from attaching to NOXs, DPP4 inhibitors, such as linagliptin, suppress ferroptosis (Xie et al., 2017).

Hydrogen groups and peroxyl radicals are formed when unstable cellular iron combines with H_2_O_2_, which further cause PUFAs to lose hydrogen atoms (PUFAs are more prone to lose hydrogen due to their pentadiene structure), forming lipid radicals L·. The resulting lipid peroxyl radical, LOO, is a byproduct of L reaction with oxygen. Since radical-trapping antioxidants (RTAs) have a high concentration of the relatively unstable O-H and N-H bonds, they can form free radicals, which may then interact with peroxyl radicals during the propagation stage to generate non-radical products that effectively stop ferroptosis (Ingold and Pratt, 2014). RTAs include ferrostatin-1 (Fer-1), liproxstatin-1 (Lip-1), alpha-tocopherol, and tetrahydrobiopterin. Fer-1 has found widespread use both *in vitro* and *in vivo*. Researchers have shown that blocking ferroptosis with Fer-1 reduces acute lung injury caused by lipopolysaccharide (Liu et al., 2020). LIP-1 has been proven to be a more durable inhibitor of ferroptosis than previous compounds, and it does not influence other forms of RCD (Li X. et al., 2019). Surprisingly, alpha-tocopherol (α-TOH) is a modest inhibitor of ferroptosis in comparison to Fer-1 or LIP-1 (Skouta et al., 2014). Tetrahydrobiotrexate, a natural free radical catcher, can also inhibit ferroptosis by increasing the CoQ_10_ reduction level.

## 4 Progress in research on ferroptosis in IVDD

### 4.1 Small molecules

**FIGURE 4:**
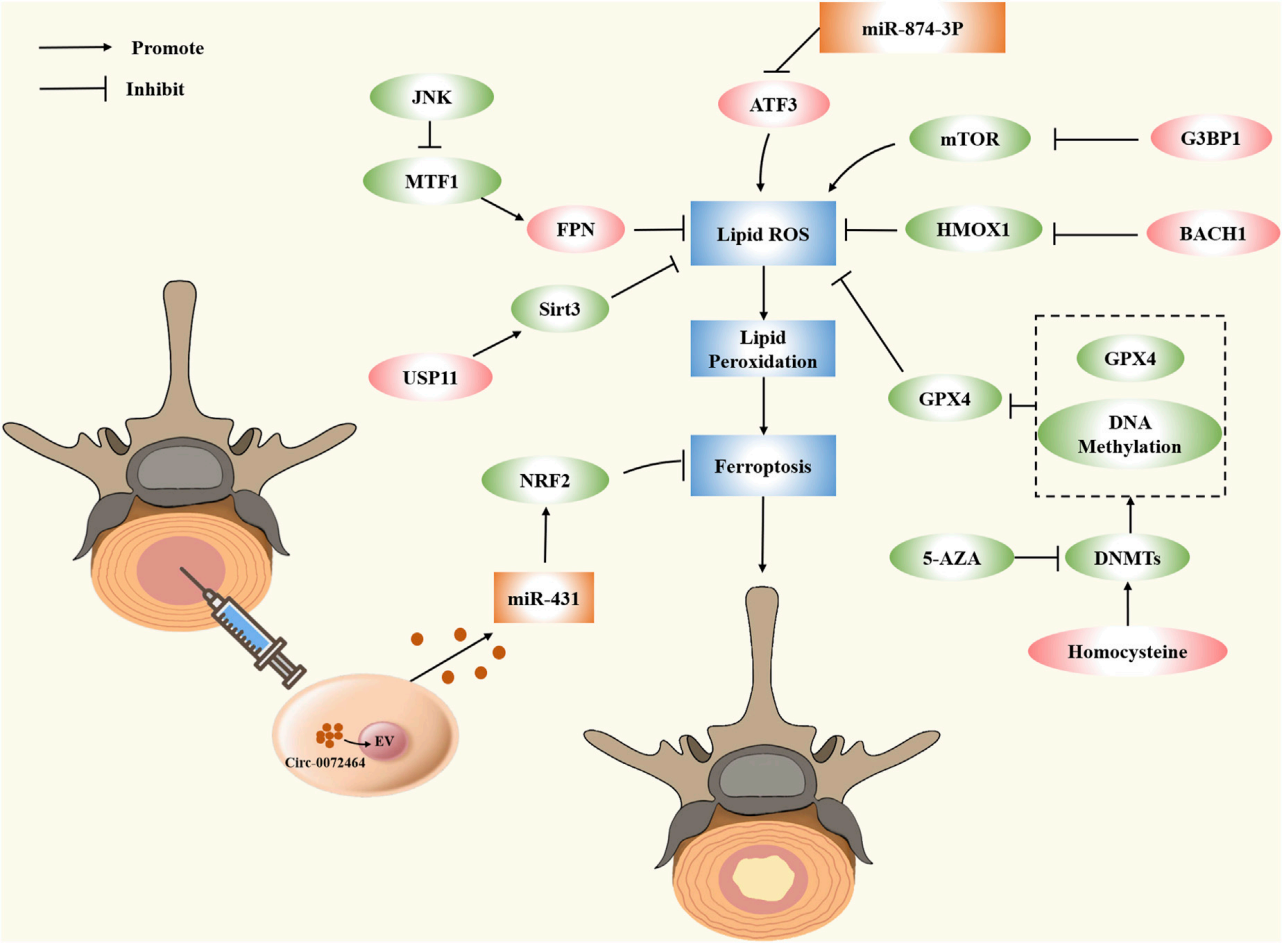
Modulating ferroptosis in IVDD by small molecule compounds and epigenetic modifications.

Increasing evidence indicates the role of small molecules in ferroptosis regulation. These proteins act as promoters and blockers by regulating the expression of target genes in metabolic and antioxidant pathways. In the description below, we summarize some small molecules associated with IVDD and present their potential applications in IVDD (Figure 4).

#### 4.1.1 FPN

Ferroportin (FPN), also known as solute carrier family 40 member (SLC40A1), is the sole iron export protein discovered to transport iron from cytoplasm to extracellular space (Drakesmith et al., 2015). FPN is strongly expressed in cells that store iron, including hepatocytes, macrophages, neurons, and duodenal gut cells, which deliver iron to the blood, to maintain the homeostasis of total iron (Donovan et al., 2005; Zhang et al., 2018). Changing the expression of FPN might result in either an iron surplus or a deficit, and the change of iron content will affect the expression of FPN (Nemeth et al., 2004; Schimanski et al., 2005; Hentze et al., 2010). Mice rely on FPN to maintain normal amounts of intracellular iron, which is essential for mitochondrial metabolism, osteoclast formation, and bone homeostasis. Increasing osteoclast genesis and reducing bone density *in vivo*, FPN depletion marginally raises iron levels in precursors and mature osteoclasts, whereas iron content decreases in precursors but not in mature cells (Wang et al., 2018b). FPN also exists in IVDD. Overproduction of iron inside cells occurs during oxidative stress due to FPN dysregulation. Iron overload leads to the generation of ferroptosis, which results in an imbalance of the extracellular matrix of nucleus pulposus cells, thereby aggravating IVDD. Rescuing FPN activity, reducing intercellular iron overload, and shielding cells from ferroptosis are all accomplished through the nuclear translocation of metal-regulated transcription factor 1 (MTF1). Bioactive substance Hinokitiol promotes nuclear translocation of MTF1 and blocks the JNK pathway, which is known to inhibit oxidative stress and restore FPN function. Lu et al. have used TBHP to establish an oxidative stress model and downregulated the expression of FPN via lowering MTF1 nuclear translocation, which leads to intracellular iron overload and ferroptosis in NPCs; thus, it is a promising therapeutic for treating IVDD associated with oxidative stress (Lu et al., 2021a).

#### 4.1.2 Homocysteine

Homocysteine (Hcy) is a sulfhydryl amino acid produced during the normal biosynthesis of methionine and cysteine. It is essential for many cellular processes, including the metabolism of methionine, nucleic acid synthesis, gene methylation, neurotransmission, and phospholipid synthesis (Mudd et al., 1985; Faeh et al., 2006; Shen et al., 2020). Evidence suggests that Hcy is associated with ferroptosis. Cao et al. demonstrated that DJ-1 preserves S-adenosine homocysteine hydrolase activity to promote the transsulfuration pathway, increasing Hcy generation and inhibiting ferroptosis (Cao et al., 2020). Cystathionine β synthase (CBS) is essential to Hcy transsulfuration. In hepatocellular carcinoma, upregulation of Hcy and subsequent ferroptosis may result from CBS inhibition (Wang et al., 2018a). Importantly, Hcy is overexpressed in musculoskeletal disorders, such as osteoarthritis, osteoporosis, and fractures (Koh et al., 2006; Fayfman et al., 2009). It has been reported that hyperhomocysteinemia promotes chondrocyte degeneration and decreases chondrocyte-mediated mineralization by boosting MMP production and the degradation of ECM associated with oxidative stress. Hyperhomocysteinemia is a major contributor to IVDD onset, according to clinical epidemiological investigations. Recently, Zhang et al. have established a new link between homocysteine and IVDD. Both the anabolic and catabolic changes of Hcy treatment are dosage-dependent. Induction of Hcy by a high-methionine diet *in vivo* can accelerate IVDD caused by a needle prick. Specifically, the researchers attribute this to homocysteine-induced oxidative stress and ferroptosis. Folic acid is a key cofactor in the methionine cycle, which reduces GPX4 methylation and protects against Hcy-induced oxidative stress and ferroptosis. Folic acid supplementation prevents the degeneration of NPCs and rescues the imbalance between anabolism and catabolism of ECM in IVDD.

#### 4.1.3 ATF3

Activation transcription factor 3 (ATF3) is a member of the ATF/CREB transcription factor family (Zhou et al., 2018). Physiologically, its functions are widespread, including the regulation of intracellular signaling pathways and cellular metabolism (Rohini et al., 2018). In several pathologies, including gastric cancer and glioma, ATF3 triggers ferroptosis (Fu D. et al., 2021; Lu et al., 2021b). ATF3 is a positive regulator of ferroptosis since it directly suppresses SLC7A11 expression in erastin-induced HT1080 cells (Wang L. et al., 2020). According to the report, ATF3 can restrain the Xc^−^ system associated with the consumption of glutathione in cells, then inhibit GPX4, and induce ferroptosis (Wang et al., 2021a). Ferroptosis significantly contributes to the worsening of spinal cord injury (SCI) development despite the fact that the underlying process through which this happens is still poorly understood. Comparing the SCI group with the sham group, bioinformatics research performed by Li et al. has revealed the top 10 hub ferroptosis genes in the subgroup, with *ATF3* ranking fourth among the differential genes of ferroptosis, which provides an idea for targeting ferroptosis to repair SCI (Li J. Z. et al., 2023). In rat NP degenerative tissues, ATF3 expression has been markedly elevated in prior research. It is possible that ATF3 is translocated into the nucleus, and its level in the nucleus is elevated in response to TBHP induction. As an important target for regulating ferroptosis, ATF3 regulates TBHP-induced ROS generation and ECM degradation in NPCs by inhibiting SLC7A11 and SOD2. Therefore, silencing ATF3 may be a potential treatment for preventing IVDD progression (Li et al., 2022).

#### 4.1.4 HO-1

Heme oxygenase-1 (HO-1) is highly expressed in lung and intestinal mucosa (Fan et al., 2019; Puentes-Pardo et al., 2020), which has become a central effector of the mammalian stress response (Ryter et al., 2006). Free heme is broken down by the vital cell-protective enzyme HO-1 into free iron, carbon monoxide (CO), and biliverdin (BV), which then can be changed into bilirubin (BR) (Wilks, 2002). The function of HO-1 in ferroptosis is contradictory. HO-1 is an antioxidant that prevents oxidative damage and inhibits ferroptosis (Adedoyin et al., 2018); when the HO-1 activity level is low to moderate, it offers cellular protection as the ROS is neutralized, and the resulting iron can enter a non-oxidophilic state. However, excessive HO-1 activation can cause ferroptosis because of elevated intracellular iron levels (Fang et al., 2019). The antioxidant response is impacted by the lowered NADPH level caused by highly activated HO-1, along with the higher iron levels that create overpowering ferritin-neutralizing effects on labile iron (Chang et al., 2018; Hassannia et al., 2018). A high oxidative iron pool promotes ferroptosis by increasing ROS and HO-1 activity and decreasing antioxidants, such as GSH (Dixon et al., 2012). HO-1 is closely related to IVDD. IVDD tissue and replicative senescent NPCs show downregulation of HO-1 expression. With HO-1 overexpression driven by a lentivirus, NPC senescence decreases, mitochondrial function is preserved, and autophagy becomes induced via a mitochondrial route (Yi et al., 2020). Chen et al. revealed that oxidative stress can inhibit the cell viability of endogenous NP-derived mesenchymal stem cells (NPMSCs), induce apoptosis, and increase ROS production. There was an uptick in HO-1 expression in the preliminary stage, but it decreased in the later stage, which could partially reverse the oxidative damage of NPMSCs by up-regulating HO-1 expression (Chen S. et al., 2020). HO-1 can inhibit the oxidative stress of IVD, thereby inhibiting inflammation, ECM metabolism, and other factors that eventually cause IVDD. Additionally, increased HO-1 expression and iron accumulation have been observed in rat IVDD models, but the specific mechanisms need to be further studied and elucidated (Zhang et al., 2021).

#### 4.1.5 G3BP1

Ras GTPase-activating protein-binding proteins 1 (G3BP1) is a multifunctional binding protein with various biological functions, which plays an important role in the regulation of cell senescence, immune response and RNA metabolism (Mao et al., 2018; Omer et al., 2020). G3BP1 is able to regulate ferroptosis. Raised G3BP1 blocking the combination of P53 and SLC7A11 gene promoter regions, less influence on SLC7A11 transcription, promote SLC7A11-GSH-GPX4 signaling pathways, inhibit ferroptosis, relieve acute liver failure (Li W. et al., 2023). Under compression pressure, G3BP1 is mainly present in lysosomes, and its dysfunction promotes the inactivation of lysophagy. Li et al. found that in nucleus pulposus cells, G3BP1 can initiate lysosomal phagocytosis to remove damaged lysosomes by regulating TSC/mTOR signaling, thereby reversing the rise of free iron and lipid peroxidation and alleviating ferroptosis (Li S. et al., 2023).

#### 4.1.6 BACH1

BTB and CNC homology 1 (BACH1) is a heme-regulated transcription factor that inhibits genes involved in iron and heme metabolism in normal cells and has the potential to form the metabolism and metastasis of cancer cells (Igarashi et al., 2021). BACH1 is involved in the regulation of various physiological processes, including oxidative stress, aging, cell cycle and mitosis (Padilla and Lee, 2021). BACH1 is an important gene in the process of ferroptosis. BACH1 has been reported to promote ferroptosis after cerebral ischemia-reperfusion injury by activating KDM4C-mediated COX2 demethylation (Zhang et al., 2023). In addition, in the acute lung injury model established by Wang et al., the deletion of BACH1 gene was found to activate the Nrf2/HO-1 signaling pathway, thereby alleviating ferroptosis (Wang et al., 2023). Surprisingly, BACH1 also plays a regulatory role in IVDD. Transcription factor BACH1 targeting GPX4 inhibits oxidative stress-induced ferroptosis to promote nucleus pulposus cell degeneration. Moreover, *in vivo* experiments showed that knockdown of BACH1 reversed the increase of ACSL4 levels and the decrease of FTH, FTL, SLC7A11 and GPX4 levels in the IVDD group. This provides a new therapeutic strategy for the treatment of IVDD (Yao et al., 2023).

#### 4.1.7 USP11

Ubiquitin specific protease 11 (USP11), located at Xp11.23, stabilizes other proteins through deubiquitination and plays an important role in regulating multiple biological processes such as cell proliferation, cancer growth and metastasis, and cancer drug resistance (Sun et al., 2019). USP11 plays an important role in the occurrence and development of ferroptosis. It has been reported that USP11 promotes autophagy activation and induces ferroptosis by deubiquitinating and stabilizing Beclin 1, thereby regulating spinal cord ischemia-reperfusion injury (Rong et al., 2022). In addition, Duan et al. found that USP11 can mediate LSH deubiquitination and activate CYP24A1 to inhibit ferroptosis in colorectal cancer (Duan et al., 2023). Zhu et al. found that Sirt3 decreased and ferroptosis occurred after the development of IVDD. USP11 can directly deubiquitinate and stabilize Sirt3. Inhibition of USP11 disrupts Sirt3 to promote oxidative stress-induced ferroptosis and aggravate IVDD (Zhu et al., 2023a).

### 4.2 Epigenetic regulation in IVD degeneration and regeneration

Epigenetic regulation involves DNA methylation, non-coding RNA and exosomes. Epigenetic regulation determines gene transcription, cell fate, and developmental processes in an organism. These mechanisms regulate ferroptosis in IVDD (Figure 3). We outline the current epigenetic regulatory mechanisms of ferroptosis in IVDD.

#### 4.2.1 Non-coding RNA

Non-coding RNAs (ncRNAs) account for nearly 60% of transcription products in human cells, including microRNAs (miRNAs), long non-coding RNAs (lncRNAs), and circular RNAs (circRNAs), which modulate cellular processes and pathways in developmental and pathological environments (Djebali et al., 2012). ncRNAs may control ferroptosis by either directly influencing important regulatory variables or indirectly influencing upstream targets, and the specific mechanism has been partially identified. According to research by Luo et al., miR-137 overexpression suppresses ferroptosis in melanoma cells by binding SLC1A5 at its 3′-UTR (Luo et al., 2018). In IVDD, ncRNA plays a similar role. Current evidence indicates that degenerative IVD demonstrates upregulation of the proinflammatory cytokine IL-6 in its cartilage tissue. Increased oxidative stress and iron retention, as well as induction of ferroptosis, in chondrocytes are results of the miR-10A-5p-mediated release of IL-6R, which acts as an IL-6 receptor (Bin et al., 2021). Because ATF3 is a direct target of miR-874-3p, as determined by bioinformatics analysis and molecular tests, this observation raises the possibility that the downregulation of miR-874-3p in IVDD may be responsible for the upregulation of ATF3 (Li et al., 2022). Circ_0072464 downregulation and miR-431 upregulation have been observed in IVDD. Circ-0072464 upregulates NRF2 expression by competitive binding of miR-431 and induces matrix synthesis and NPC proliferation, causing a reduction in IVDD and the suppression of NPC ferroptosis (Yu et al., 2022). This finding opens the door to future treatment efforts aimed at reducing IVDD by inhibiting NPC ferroptosis. Additionally, when it comes to SCI, ncRNA is also commonly employed. MiR-672-3p inhibits ferroptosis through the FSP1 pathway, plays a neurorestorative role *in vivo* and *in vitro*, and improves motor function in SCI rats (Wang et al., 2022). Furthermore, lncGm36569, as a competitive RNA of miR-5627-5p, induces the upregulation of FSP1 and inhibits ferroptosis in neuronal cells, thereby attenuating neuronal dysfunction (Shao et al., 2022).

#### 4.2.2 DNA methylation

DNA methylation, put forward in 1944 by Avery (Avery et al., 1944)., is an epigenetic mechanism. The DNA methyltransferase (Dnmts) family is responsible for catalyzing DNA methylation, which occurs when S-adenosine methionine (SAM) methyl groups are transferred to the fifth carbon of cytosine residues to produce 5 mC. DNA methylation patterns in the genome undergo dynamic changes throughout development as a consequence of *de novo* DNA methylation and demethylation. Gene expression may be controlled by DNA methylation, which either recruits proteins participating in gene repression or blocks transcription factors connecting to DNA (Moore et al., 2013).

DNA methylation is widely used in orthopedics. According to research by Yang et al., THAP9-AS1 is highly expressed in osteosarcoma, where it increases methylation of the SOCS3 promoter, thereby promoting carcinogenesis while, at the same time, reducing ROS generation through the JAK2/STAT3 signaling pathway (Yang S. et al., 2021). Additionally, in SCI rats after treadmill exercise, DNA methylation is enhanced, and functional recovery is promoted. Consequently, we draw the conclusion that epigenetic modifications in the motor cortex may play a role in the functional benefits obtained by exercise (Davaa et al., 2021). DNA methylation has been significantly associated with ferroptosis. Head and neck cancer cells with DNA hypermethylation at *CDH1* gene promoter reduce the production of e-cadherin (*CDH1*-encoded) and are more sensitive to iron (Lee J. et al., 2020). Gene expression can be inhibited by methylation transfer, which can also cause methylation. Methylase expression of DMNT1, DMNT3a, and DMNT3b was increased by Hcy in a study by Zhang et al. NPCs treated with Hcy have extensive methylation of GPX4 promoter. Enhanced GPX4 methylation upregulates oxidative stress and ferroptosis, thereby promoting ECM metabolic disorder in NPCs. Folic acid is a crucial coenzyme that inhibits the elevated methylase stimulated by Hcy. Gene and protein expression of GPX4 are both suppressed by methylation and recovered by methylase inhibitors, such as 5-AZA and folic acid (Zhang X. et al., 2020), suggesting a potential application of folic acid in IVDD. More research is necessary to determine whether DNA methylation also impacts additional genes involved in ferroptosis.

#### 4.2.3 Extracellular vesicles

Exosomes and microvesicles are released from cells, play important roles in cell-to-cell communication and disease development, and serve as biomarkers due to mRNAs, miRNAs, lipids, and proteins they carry (Mead and Tomarev, 2020). Recently, extracellular vesicles have been increasingly used in ferroptosis for their therapeutic value. Lin et al. found that endothelial progenitor cells-secreted extracellular vesicles (EPC-EVs) inhibited FSP1 by delivering miR-199a-3p. As a result, endothelial ferroptosis was suppressed, and atherosclerosis development was slowed (Li L. et al., 2021). Recently, the use of EVs released by MSC as a treatment for IVDD has gained popularity (Sun Y. et al., 2021). BMSC-EV-loaded CIRC 0072464 suppresses NPC ferroptosis via up-regulating miR-431-mediated NRF2 to ameliorate IVDD, offering a possible therapeutic target for IVDD (Yu et al., 2022).

## 5 Possible therapeutic intervention

Ferroptosis is a promising future research topic and might have important therapeutic applications. Ferroptosis has a connection to the occurrence and prognosis of numerous illnesses, such as cancer, hematologic diseases, neurodegenerative diseases, and heart diseases. Additionally, many published studies now point in the direction of ferroptosis as a viable option for treating a wide range of orthopedic disorders. The prognosis for osteosarcoma, the most frequent primary malignant bone cancer, is not optimistic. The major cause of osteosarcoma not responding to therapy and subsequent relapse is drug resistance. By blocking the STAT3/Nrf2/GPX4 signaling pathway, osteosarcoma cells become more susceptible to cisplatin. This finding demonstrates a unique strategy for increasing drug sensitivity in osteosarcoma by either ferroptosis inducers or STAT3 inhibitors (Liu and Wang, 2019). Furthermore, synergistic growth inhibition in hypoxic osteosarcoma has been achieved by the induction of ferroptosis and substantial GPX4 downregulation using ultrasound-activated doxorubicin (DOX)-Fe(VI)@HMS-HE-PEG (DFHHP) nanoparticles (Fu J. et al., 2021). SCI is a disorder of the neurological system caused by a sudden injury, resulting in varying degrees of motor, sensory, and autonomic dysfunction. Ferroptosis inhibitor U0126 blocks the RAS/RAF/ERK pathway, which restores SCI and decreases glial scar formation in injured regions. This has also been achieved by lowering astrocyte proliferation, protecting neurons, and promoting axonal regeneration (Table 3).

**TABLE 3:** Interventions for IVDD ferroptosis.

Intervention	Mechanism	Result	References
SiRNA	Targeting ferroportin and Iron homeostasis	Inducing ferroptosis of IVDD	Lu et al. (2021a)
Homocysteine	Enhancing methylation of GPX4	Inducing ferroptosis of IVDD	Zhang et al. (2020b)
Silencing ATF3	Restoring SLC7A11 and SOD2	Inhibiting ferroptosis of IVDD	Li et al. (2022)
G3BP1	Coordinating TSC/mTOR complex to initiate lysophagy	Inhibiting ferroptosis of IVDD	Li et al. (2023b)
BACH1	Regulating HMOX1/GPX4 signaling	Inducing ferroptosis of IVDD	Yao et al. (2023)
USP11	Deubiquitinating and stabilizing Sirt3.	Inhibiting ferroptosis of IVDD	Zhu et al. (2023a)
Hesperidin	Regulating Nrf2/NF-κB signaling	Inhibiting ferroptosis of IVDD	Zhu et al. (2023b)
Non-coding RNA	Inhibiting miR-10a-5p/IL-6R signaling	Inhibiting ferroptosis of IVDD	Bin et al. (2021)
BMSC-EVs	Activating circ_0072464/miR-431/NRF2 axis	Inhibiting ferroptosis of IVDD	Yu et al. (2022)

Moreover, ferroptosis points the way to the clinical treatment of IVDD. The formation of new blood vessels is a characteristic of IVDD. Yasuma et al. first observed neovascularization in the herniated NP of LDH patients by histological staining in 1993 (Yasuma et al., 1993). It has been recently proposed that angiogenesis promotes tissue degradation following disc damage and may be a healing mechanism (Xiao et al., 2020). Vascularized granulation tissue produces inflammatory cytokines that stimulate nociceptors in the protruding NP, leading to further exacerbation of clinical symptoms (Yasuma et al., 1993). Previous studies have shown that hemoglobin is released from red blood cells oozing from immature capillaries in atherosclerotic lesions and human tumors (Nagy et al., 2010). The oxidation of hemoglobin to ferriyl hemoglobin then results in the emission of iron porphyrins, leading to an increase in intracellular iron content (Chifman et al., 2014). Matrix-assisted laser desorption-ionization time of flight mass spectrometry (MALDI-TOF MS) was used to analyze clinically herniated and nonherniated NP; the study found a significant increase in hemoglobin and heme signaling and a decrease in ferroptosis markers GPX4 and glutathione in herniated NP. Therefore, herniated NP with neovascularization may expose tissue to high amounts of heme, which may trigger cytotoxicity and ferroptosis and accelerate progressive degeneration. These findings aid in the study of degenerative illnesses and will lead to novel approaches for the conservative treatment of patients with LDH, for instance, angiogenesis intervention (Shan et al., 2021).

Ferritin autophagy is a property of intracellular iron concentration and selective ferritin degradation. Serum iron metabolism parameters, particularly serum ferritin (SF), have been the subject of research on IVDD by Guo et al. Correlation analysis has shown that intervertebral disc degeneration was significantly associated with SF but not with serum iron (SI), transferrin saturation (TS), unsaturated iron binding capacity (UIBC), and total iron binding capacity (TIBC). The concentrations of SF were surprisingly greater in patients with low-severe IVDD compared to those with high-severe IVDD in the single-disc grade. According to the receiver operating characteristic curve, patients with SF levels below 170.5 ng/mL exhibited advanced disc degeneration. Therefore, SF is inversely associated with IVDD severity, suggesting a novel approach to the clinical prediction of IVDD severity.

## 6 Conclusion and prospects

With the help of ongoing studies, IVDD has transformed from a debilitating condition to a treatable one. While current IVDD treatments, such as medication and surgery, are effective, they also come with drawbacks, such as a high recurrence rate, excessive bleeding, and other complications. Therefore, improving IVDD is a difficult problem for clinicians.

Ferroptosis has received a lot of attention, and great progress has been recently achieved in its research. In this article, we provided a summary of the cellular morphology, pathogenesis, and regulation of ferroptosis. Iron metabolism, lipid peroxidation, ROS accumulation, GPX4 regulation, and FSP1-mediated pathway are a few of the processes involved in ferroptosis. However, the study of ferroptosis in IVDD is just beginning and there are still many unanswered questions. First, current studies of ferroptosis in IVDD are only scratching the surface of the results, and it is unclear exactly what causes it and what the target molecules and associated signaling pathways are. Second, many researchers have only studied ferroptosis in cell and animal models, and there is a lack of effective clinical evidence. Subsequently, ferroptosis in IVDD have been studied in nucleus pulposus cells, while other intervertebral disc cells, such as annulus fibrosus cells and endplate chondrocytes, have been poorly studied. In addition, many long non-coding RNAs and circular RNAs have been found to affect ferroptosis in IVDD, indicating that molecular mechanisms at the post-recording level are important in regulating cellular ferroptosis, and more small RNAs related to ferroptosis need to be explored in the future. Additionally, current models of ferroptosis in IVDD are primarily caused by oxidative stress and inflammation. Factors such as hypoxia and acidic microenvironment need to be confirmed whether they can be used as ferroptosis models in IVDD. Finally, as we all know, mixed cell death has become a hot topic. At present, autophagy is closely related to ferroptosis. A large number of literatures have proved that ferroptosis is an autophagy-dependent cell death (Zhou et al., 2020). It is also involved in the intervertebral disc field. Li et al. found that G3BP1 coordinates TSC/mTOR complex activation of lysophagy, clearance of damaged lysosomes, and inhibition of ferroptosis. However, there are few published literatures, and the relationship between autophagy and ferroptosis in intervertebral disc has not been clarified. Furthermore, whether other forms of cell death, such as apoptosis, interact with ferroptosis also needs to be further explored.

## Figures and Tables

**Table:** 

**IVD**	Intervertebral disc
**IVDD**	Intervertebral disc degeneration
**RCD**	Regulated cell death
**ROS**	Reactive oxygen species
**LBP**	Low back pain
**ECM**	Extracellular matrix
**Col II**	Type II collagen
**Col I**	Type I collagen
**MMPs**	Metalloproteinases
**ER**	Endoplasmic reticulum
**PRMPs**	Pathogen-related molecular patterns
**DAMPs**	Damage-related molecular patterns
**PRR**	Pattern recognition receptor
**GSDMD**	Gasdermin-D
**NPCs**	Nucleus pulposus cells
**RIP**	Receptor interacting protein
**STEAP3**	Six-transmembrane epithelial antigen of prostate family member 3
**SLC11A2/DMT1**	Member 2 of solute carrier family 11
**LIP**	Labile iron pool
**FTH1**	Ferritin heavy chain 1
**FTL**	Ferritin light chain
**FAs**	Fatty acids
**LOOHs**	Lipid hydroperoxides
**MDA**	Malondialdehyde
**4HNE**	4-hydroxynonenal
**PUFAs**	Polyunsaturated FAs
**AA**	Arachidonic acid
**PLs**	Phospholipids
**LOX**	lipoxygenase
**PE**	Phosphatidylethanolamine
**PEBP1**	Phosphatidylethanolamine binding protein 1
**OMM**	Outer membrane
**ACSL4**	Acyl-CoA synthetase long-chain family 4
**TZD**	Thiazolidinediones
**ROSI**	Rosiglitazone
**MUFAs**	Monounsaturated FAs
**ACSL3**	Acyl-CoA synthase long-chain family member 3
**GSH**	Glutathione
**SLC7A11**	Solute carrier family 7 member 11
**SLC3A2**	Solute carrier family 3 member 2
**GCL**	Glutamate–cysteine ligase
**PLOH**	Phosphatidylcholine
**TMZ**	Temozolomide
**NAC**	N-Acetylcysteine
**CoQ**	Coenzyme Q
**MVA**	Mevalonate
**IPP**	Isopentenyl pyrophosphate
**GGPS**	Pyrophosphate synthase
**SQS**	Squalene synthase
**VDAC2/3**	Voltage-dependent anion channel 2/3
**GTP**	Guanosine triphosphate
**GCH1**	Guanosine triphosphate cyclic hydrolase 1
**BH4**	Tetrahydrobiotrexate
**DHFR**	Dihydrofolate reductase
**NOX**	NADPH oxidases
**DPP4**	Dipeptidyl-peptidase 4
**RTAs**	Radical-trapping antioxidants
**Fer-1**	Ferrostatin-1
**Lip-1**	Liproxstatin-1
**α-TOH**	Alpha-tocopherol
**DFO**	Deferoxamine
**DFX**	Deferasirox
**DFP**	Deferiprone
**BHT**	Butylated hydroxytoluene
**FPN**	Ferroportin
**SLC40A1**	Solute carrier family 40 member
**MTF1**	Metal-regulated transcription factor 1
**Hcy**	Homocysteine
**CBS**	Cystathionine β synthase
**ATF3**	Activation transcription factor 3
**SCI**	Spinal cord injury
**HO-1**	Heme oxygenase-1
**G3BP1**	Ras GTPase-activating protein-binding proteins 1
**BACH1**	BTB and CNC homology 1
**USP11**	Ubiquitin specific protease 11
**CO**	Carbon monoxide
**BV**	Biliverdin
**BR**	Bilirubin
**NPMSCs**	NP-derived mesenchymal stem cells
**ncRNAs**	Non-coding RNAs
**miRNAs**	MicroRNAs
**lncRNAs**	Long non-coding RNAs
**circRNAs**	Circular RNAs
**Dnmts**	DNA methyltransferase
**SAM**	S-adenosine methionine
**EPC-EVs**	Endothelial progenitor cells-secreted extracellular vesicles
**MALDI-TOF MS**	Matrix-assisted laser desorption-ionization time of flight mass spectrometry
**SF**	Serum ferritin
**SI**	Serum iron
**TS**	Transferrin saturation
**UIBC**	Unsaturated iron binding capacity
**TIBC**	Total iron binding capacity
